# Prediction of Recurrence and Rupture Risk of Ruptured and Unruptured Intracranial Aneurysms of the Posterior Circulation: A Machine Learning-Based Analysis

**DOI:** 10.3390/diagnostics15182365

**Published:** 2025-09-17

**Authors:** Martin Növer, Hanna Styczen, Ramazan Jabbarli, Philipp Dammann, Martin Köhrmann, Tim Hagenacker, Christoph Moenninghoff, Michael Forsting, Yan Li, Isabel Wanke, Aydin Demircioğlu, Cornelius Deuschl

**Affiliations:** 1 Department of Anaesthesiology and Intensive Care Medicine, University Hospital Essen, Hufelandstrasse 55, 45147 Essen, Germany; 2 Institute of Diagnostic and Interventional Radiology and Neuroradiology, University Hospital Essen, Hufelandstrasse 55, 45147 Essen, Germany; cornelius.deuschl@uk-essen.de (C.D.); 3 Department of Neurosurgery, University Hospital Essen, Hufelandstrasse 55, 45147 Essen, Germany; 4 Department of Neurology, University Hospital Essen, Hufelandstrasse 55, 45147 Essen, Germany; 5 Department of Radiology, Neuroradiology and Nuclear Medicine, Johannes Wesling University Hospital, Hans-Nolte-Strasse 1, 32429 Minden, Germany; 6 Swiss Neuroradiology Institute, Bürglistrasse 29, 8002 Zürich, Switzerland

**Keywords:** intracranial aneurysm, basilar artery, recurrence, rupture, machine learning

## Abstract

**Background**: Intracranial aneurysms of the posterior circulation are of particular clinical significance due to their higher risk of rupture-associated morbidity and mortality compared to anterior circulation aneurysms. Moreover, they exhibit an increased tendency for recurrence, posing challenges for long-term management. The purpose of this study is to identify key risk factors and define criteria for the early detection of high-risk aneurysms with a machine learning-based analysis. **Methods**: This study employs machine learning (ML), which, unlike traditional statistical methods, can detect complex, previously unrecognized patterns without predefined hypotheses to predict recurrence and rupture in patients with intracranial aneurysms of the posterior circulation. A total of 229 patients were retrospectively screened (2008–2020), and the data set was analyzed using ML algorithms. To avoid bias, a 10-fold cross-validation was employed, and the model performing best in terms of the Area Under the Curve (AUC) was selected. In addition, the sensitivity, specificity, and accuracy of the model were computed as secondary metrics. **Results**: A total of 229 patients were included, with over 70% being female, older than 50 years, and diagnosed with arterial hypertension. The most significant predictors of aneurysm recurrence identified by the ML model (AUC of 0.74 with a sensitivity of 0.76, a specificity of 0.70, and an accuracy of 0.76) were age, aneurysm size, arterial hypertension, and a history of nicotine consumption. The DeLong test confirmed that the ML model performed significantly better than random classification with an AUC of 0.5 (*p* < 0.001). Further analysis revealed that the presence of multiple aneurysms and localization at the basilar artery were independent risk factors for early recurrence within six months. For aneurysm rupture, key predictive features included advanced age, basilar artery localization, atherosclerosis, irregular aneurysm morphology, and familial predisposition. **Conclusions**: ML algorithms identified several risk factors for recurrence and rupture of intracranial aneurysms of the posterior circulation, aligning with previously established risk factors. These findings are intended to serve as a basis for further research in clinical use and prospective studies.

## 1. Introduction

Aneurysms of the posterior circulation are of particular interest, as compared to aneurysms of the anterior circulation. They have an increased risk of rupture and recurrence and their location is directly associated with a poorer treatment outcome [1]. If untreated, intracranial aneurysms may cause subarachnoid hemorrhage (SAH) and fatal outcomes. Their etiology remains multifactorial and incompletely understood [2]. Posterior circulation aneurysms are less common but associated with higher recurrence rates and worse prognoses upon rupture [3]. Established risk factors include genetic predisposition, atherosclerosis, arterial hypertension, nicotine use, and excessive alcohol consumption (>150 g/week) [4,5,6,7,8]. It has also been proven that the probable risk of rupture is directly related to the size of the aneurysm [9]. Studies from Japan and Finland show higher event rates for small aneurysms, meaning that a genetic component for an increased risk of rupture cannot be ruled out [2,10].

Clinically, unruptured aneurysms may be asymptomatic or cause neurological deficits due to mass effect, while rupture typically presents with acute severe headache [11]. SAH has a high mortality and morbidity, with only one-third of patients surviving without neurological impairment [12].

Machine learning techniques have become an important statistical modeling tool because they can determine associations in a purely data-driven way and do not depend on pre-specified hypotheses. The main task of machine learning is to develop models that independently learn patterns and regularities from given data sets. The knowledge gained in this way is generalized and enables predictions to be made for new data sets [13]. To date, there is no further study on the prediction of recurrences and ruptures in patients with aneurysms of the posterior circulation with ML techniques.

Machine learning has already been proven to detect and differentiate ruptured and unruptured intracranial aneurysms in general [14,15]. The currently available studies agree that the use of ML algorithms is superior to human diagnosis in detecting even small intracranial aneurysms (<7 mm diameter) [16]. Machine learning can also be used to identify risk factors for diseases. In a machine learning—based study, for example, various risk factor combinations were shown to predict SAH [17].

The aim of this study is to present the recurrence and rupture rates of endovascularly treated aneurysms of the posterior circulation with an evaluation and detection of risk factors associated with recurrence and rupture. This should help individuals monitor patients with such risk factors more closely, detect the development of aneurysms or their recurrence at an earlier stage, and prevent clinically dangerous and prognostically unfavorable courses. The use of machine learning as a statistical tool should open up further possibilities for future research, such as the efficient and reliable analysis of larger, more complex data sets. 

## 2. Materials and Methods

This work is a retrospective single-center study analyzing patients with intracranial aneurysms of the posterior circulation treated endovascularly between 2008 and 2020. The patient files and radiological images were inspected and the anamnesis, imaging diagnostics, and the clinical course of these patients were documented. Furthermore, a statistical analysis of the data sets was performed using machine learning. To independently learn patterns and regularities from given data sets, this method can be used. It is not dependent on a predetermined hypothesis.

### 2.1. Patient Population

**Figure 1:**
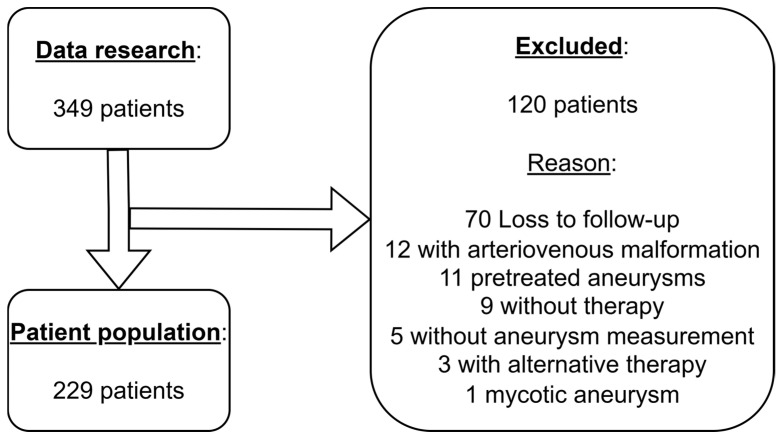
Selection of patient population.

During the observation period from January 2008 to July 2020, the data of all patients who underwent endovascular treatment for one or more intracranial aneurysms of the posterior circulation were examined retrospectively. These included aneurysms of the basilar artery, posterior cerebral artery, posterior communicating artery, and the intracranial parts of the vertebral artery (V4), superior cerebellar artery, anterior inferior cerebellar artery, and posterior inferior cerebellar artery. Initially, the data of 349 patients were documented and analyzed. As shown in Figure 1, in the end, 229 patients with endovascularly treated aneurysm were included in the study.

### 2.2. Data Collection

The Radiology Information System (Centricity RIS-i 6.0, GE HealthCare Technologies, Chicago, IL, USA), which can be used for data management, diagnostic reporting, and archiving, and the Picture Archiving and Communication System (Centricity PACS, GE Healthcare), which is used for processing, managing, and archiving medical images and data, served as the primary sources of information. With the help of the Medico software program (CGM Medico, CGM Clinical Europe GmbH, Koblenz, Germany), a hospital information system, it was possible to track patients’ anamnestic characteristics.

### 2.3. Criteria of Medical Evaluation

Patient-related characteristics such as name, gender, and age at the time of the intervention were extracted from the electronic patient file, whereby the name was anonymized for further data processing. Important previous diseases such as arterial hypertension, diabetes mellitus, and atherosclerosis, as well as cardiovascular risk factors such as nicotine consumption and Body Mass Index (BMI), were documented [7,18]. It was evaluated whether a familial disposition or genetic diseases were present [4,5].

Furthermore, we extracted aneurysm-related characteristics such as the presence of multiple aneurysms, localization, configuration, and size of the aneurysms from the Radiology Information System (RIS) and Picture Archiving and Communication System (PACS). Anatomical vessel anomalies and the occurrence of intracranial hemorrhage due to aneurysm rupture were documented.

During a mean follow-up period of 20.2 months (1 day–126 months), we were able to track the morphologic course of the aneurysms.

Based on the medical reports, the clinical course could be traced. Both the Hunt and Hess (H & H) classification and the Glasgow Coma Scale (GCS) were used to assess the initial clinical condition on hospitalization.

### 2.4. Image Analysis

All patients initially received a diagnostic Digital Subtraction Angiography (DSA), in which the diagnosis, localization, size, and configuration of one or more aneurysms were determined. Following an interdisciplinary decision in favor of interventional therapy, an emergency or elective therapeutic DSA was performed, depending on whether an aneurysm rupture was present or not.

**Figure 2:**
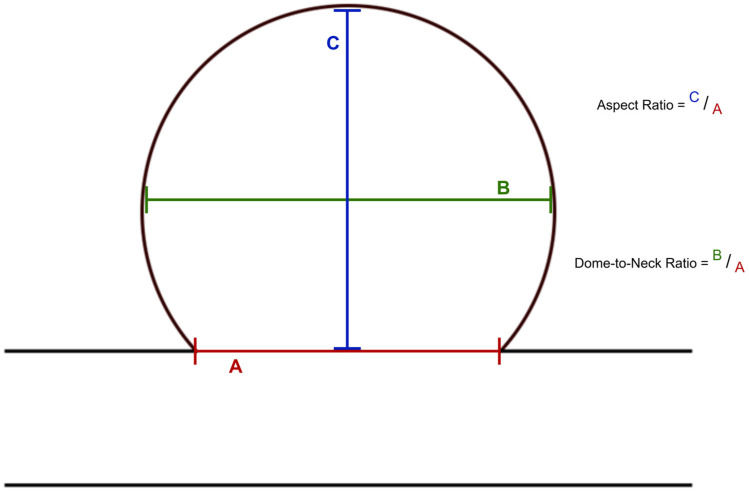
Aneurysm measurement.

The size classification of the aneurysms is based on the UCAS [19]. In order to standardize the measurements, the aneurysm size was measured in three dimensions and the largest diameter was selected in each case. Care was taken to ensure that all measurements were performed by one operator in order to obtain standardized comparability. In addition to the neck width, the measurements included both the dome height and the dome width. These parameters were used to determine the dome-to-neck ratio and the aspect ratio, as shown in Figure 2. The neck width, dome-to-neck ratio, and aspect ratio have already been described as significant factors that correlate with treatment success [20,21].

### 2.5. Machine Learning

For the analysis of the data, ML algorithms were employed. Their main advantage over traditional statistical methods is that they operate without predefined hypotheses, enabling them to uncover complex and previously unrecognized patterns. By prioritizing prediction, they can enhance decision-making and forecasting, making them particularly valuable for improving outcomes in clinical routines.

Application of machine learning usually proceeds in several steps. The data is first pre-processed. Then, feature selection methods are used to remove redundant and irrelevant features. This step can improve the performance and the interpretability of the resulting model. Then, a classifier is used to find an underlying relationship between the data and the outcome. However, since it is not known from the outset which feature selection method and which classifier is best suited for a particular data set, several methods are tested in machine learning. Since machine learning is data-driven, there is a high risk that the model will overfit the training data and thus exhibit a strong bias, which can potentially lead to deteriorated performance when the model is applied on new data in a clinical context. Therefore, it is vital that the performance of the model is determined using an appropriate validation scheme on previously unseen data.

In this study, nine commonly used feature selection algorithms were employed for the model, as listed in Table 1. Since these methods do not select features directly, but rather score them according to their relevance, it was necessary to determine how many of the highest-scoring features should be used. Four values were tested: n = 4, 8, 12, and 16. Additionally, modeling was also performed without any feature selection to serve as a baseline.

For modeling, five classifiers were evaluated: Naïve Bayes [22], Logistic Regression [23], Neural Networks [24], Random Forests [25] and non-linear Support Vector Machine (SVM) with a Radial Basis Function (RBF) kernel [26]. The classifiers are shown in Table 2.

“Naïve Bayes” is a probabilistic classifier that models the class membership based on Bayes’ theorem and the assumption of feature independence. Given features x_1_, …, x_n_, the probability of a class C is modeled by

$$P\;\left(C\;\right|x_{1},\;\ldots ,\;x_{n})=P(C)\;\prod_{i=1}^{n}P(x_{i}\;|\;C).$$

This assumption makes Naïve Bayes computationally efficient and often effective even when the independence assumption is not strictly true. However, being a rather simple classifier, Naïve Bayes can be understood as a baseline.

“Logistic regression” is a linear classification method that models the probability of the binary outcome probability using the logistic function

$$P\left(x_{1},\;...\;\;x_{n}\right)=\frac{1}{1+e^{-\left(\beta_{0}+\beta_{i}x_{i}\right)}},$$

where the parameters β are fitted by maximizing the likelihood.

“Neural networks” extend linear models by stacking layers of neurons, which subsequently transform the input data non-linearly into a prediction. Each layer applies a linear transformation followed by a non-linear activation f , yielding

$$h^{\left(l\right)}=f\left(W^{\left(l\right)}h^{\left(l-1\right)}+b^{\left(l\right)}\right).$$

Here, $W^{\left(l\right)}$ denotes the weight matrix of layer l , $b^{\left(l\right)}$ is the bias vector, $h^{\left(l-1\right)}$ is the input from the previous layer, $h^{\left(0\right)}$ is the input data, and $h^{\left(l\right)}$ is the output of the current layer after applying the non-linear function.

“Random forests” are ensemble methods that combine the predictions of multiple decision trees. Each tree is trained on a bootstrap sample of the data, and at each split, only a random subset of the features is considered. Predictions of the trees are then aggregated by majority vote. This process reduces variance compared to a single tree and improves generalization.

Finally, “Support Vector Machines” aim to find a decision boundary in the form of a hyperplane $w^{T}x\;+\;b\;=\;0$ that maximizes the margin between the two classes. This leads to an optimization problem of the form

$$\frac{1}{2}\;\;{\left||\;w\;|\right|}^{2}{\;such\;that\;y_{i}\;(w}^{T}x_{i}+b)\;\geq \;1.$$

By employing a kernel, SVMs can implicitly project data into high-dimensional spaces and thus find non-linear optimal decision boundaries.

Modeling proceeded using 10-fold stratified cross-validation. The data was first split into ten folds of similar size. Each of the folds was then set aside once for validation. The models were then trained on the pooled data from the other nine folds.

Training was performed using a simple grid search. In this approach, a set of plausible values for the main hyperparameter of each classifier was defined. A hyperparameter is a setting that controls how the model is trained. For example, the regularization parameter C in an SVM controls how the model handles noise in the training data. If C is small, the classifier tolerates some misclassifications, effectively ignoring small irregularities that do not follow an overall pattern. In contrast, a large C forces the classifier to correctly classify all data, which can lead to highly complex decision boundaries. Choosing the optimal C is necessary to optimize the overall performance of the classifier. Grid search then systematically evaluates each possible combination of the selected hyperparameters. For each combination of feature selection methods and classifiers with chosen hyperparameters, a model was trained on the training folds of the cross-validation splits. The optimized hyperparameters are listed in Table 2. For the neural network, the number of layers were fixed to three, while the number of neurons in each of the layers was considered a hyperparameter. The resulting models were then evaluated on the left-out validation fold. This process was repeated across folds to reduce random variability, ensuring that each classifier was fairly compared under optimized hyperparameters. Care was taken to ensure that all modeling steps were only performed on the training folds to ensure that the validation was unbiased, which can happen due to data leakage [25].

The predictions across all folds for each combination of feature selection and classifier were then pooled to obtain a single Receiver Operating Characteristics (ROC) curve. The main evaluation metric was chosen to be the Area Under the Curve (AUC), which measures the ability of the model to discriminate between the two classes across all classification thresholds. In addition to AUC, sensitivity (true positive rate), specificity (true negative rate), and accuracy were computed as secondary performance metrics. These are defined as follows:

$$\mathrm{Sensitivity}\;=\;\frac{TP}{TP\;+\;FN},\;\mathrm{Specificity}\;=\;\frac{TN}{TN\;+\;FP},\;\mathrm{Accuracy}\;=\;\frac{TP\;+\;TN}{TP\;+\;TN\;+\;FP\;+\;FN}$$

where TP, TN, FP, and FN represent true positives, true negatives, false positives, and false negatives, respectively. These were computed at the optimal classification threshold determined by maximizing the Youden Index J, defined by J = Sensitivity + Specificity − 1. The best-performing model in terms of AUC was selected as the final model.

The relevance of the features for the final model was then calculated using the permutation importance method [25]. In a linear model, the importance of a single feature can be measured by the standardized regression coefficient (or the associated *p*-value). The permutation importance method is a generalized, model-independent version of this value, estimating feature importance by measuring the increase in model error after shuffling the feature values. For comparison, the Shapley Additive explanation (SHAP) values were computed and visualized using bee swarm plots. Unlike permutation importance, SHAP values consider both individual feature effects and interaction between features and could therefore offer a more comprehensive explanation of model predictions.

Modeling with machine learning was performed with the scikit-learn package in Python 3.7.

### 2.6. Descriptive Statistics

Data are presented either as a percentage or as mean values with standard deviations (SD). Bootstrapping was used to calculate the 95% confidence intervals (CI), while a DeLong test was used to compare the AUC of the different ROC curves. No correction for multiple testing was applied.

## 3. Results

Table 3 summarizes the patient population, which consisted of a total of 229 patients who underwent endovascular treatment for at least one aneurysm of the posterior circulation.

All aneurysms were located in the posterior cerebral circulation. The aneurysms were measured in dome width, dome height, and neck width, and the dome-to-neck ratio and aspect ratio were calculated, as shown in Table 4.

According to the classification of the UCAS, 90 patients (39.3%) had small aneurysms of <5 mm, 101 (44.1%) had a medium-sized aneurysm of 5–10 mm, 37 (16.2%) had a large aneurysm of 10–25 mm, and 1 (0.4%) had a giant aneurysm ≥25 mm. The distribution of neck size was mostly balanced. A total of 111 individuals (48.5%) of the patient population had a wide aneurysm neck ≥4 mm. The dome-to-neck ratio was < 2 in 180 patients (78.6%) and ≥2 in 49 (21.4%). Of all the aneurysms, 96 (41.9%) were lobulated and therefore irregularly configured. Of these, 53 (55.2%) were ruptured and 43 (44.8%) were unruptured aneurysms. Of the 229 patients, 75 (32.8%) developed a recurrence during the observation period. Of these 75 recurrences, 46 (61.3%) occurred within the first six months, 14 (18.7%) between six and twelve months, and 15 (20.0%) after over twelve months, after treatment.

### 3.1. Recurrence

In all cases, the Bhattacharyya feature selection method combined with a neural network as a classifier performed with the highest AUC. The complete results of the cross-validation can be found in the Supplementary Materials.

**Figure 3:**
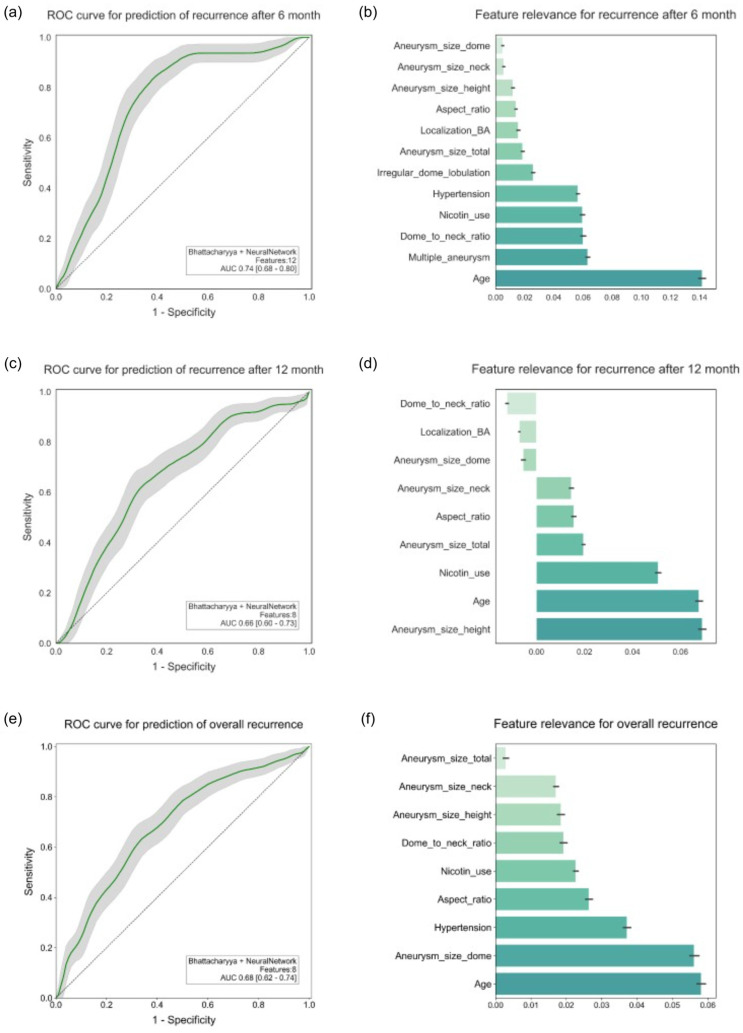
ROC curves and feature relevance for prediction of recurrence.

Figure 3a shows that the best model for predicting recurrence after six months achieved an AUC of 0.74 (95% CI: 0.68–0.80) with a sensitivity of 0.76, a specificity of 0.70, and an accuracy of 0.76. The DeLong test showed that the model was significantly better than a random estimate, with an AUC of 0.5 (*p* < 0.001). The most important feature of this model according to the permutation test was age, followed by the occurrence of multiple aneurysms, the dome-to-neck ratio, and the presence of risk factors such as current or past nicotine consumption and arterial hypertension. The feature importance obtained using SHAP did not largely deviate; notably age, arterial hypertension, and dome-to-neck ratio were consistent. Furthermore, an irregular dome configuration and aneurysmal localization at the basilar artery were important features, which is illustrated in Figure 3b.

Figure 3c shows that the best model for predicting recurrence after twelve months performed slightly worse, achieving an AUC of 0.66 (95% CI: 0.62–0.74; *p* = 0.004). The sensitivity and specificity were 0.63 and 0.68, respectively, while the accuracy was 0.70. The dome height of the aneurysm, age, and the presence of the risk factor nicotine consumption, were the most important characteristics, as shown in Figure 3d. Compared to SHAP, no correlation with nicotine consumption could be demonstrated.

The prediction of all recurrences was comparably good, with an AUC of 0.68 (95% CI: 0.68–0.74; *p* < 0.001). Figure 3e demonstrates that the model showed a sensitivity of 0.65 and a specificity of 0.67, as well as an accuracy of 0.69. The model shows age and aneurysm dome width are the two most important features, followed by the presence of arterial hypertension, as well as the aspect ratio. SHAP also showed age, aneurysm size, and, additionally, dome-to-neck ratio as important characteristics. Figure 3f provides an illustration of this.

### 3.2. Rupture

**Figure 4:**
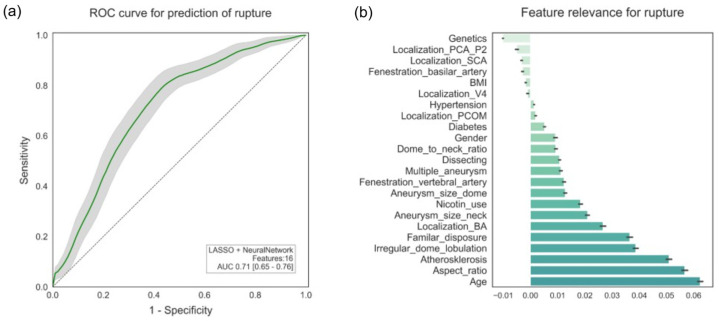
ROC curve and feature relevance for rupture.

For the prediction of rupture, the best model consisted of “LASSO” as feature selection, with 16 features, and “neural network” as classifier. Figure 4a shows that it achieved an AUC of 0.71 (95% CI: 0.65–0.76; *p* < 0.001), a sensitivity of 0.72, a specificity of 0.65, and an accuracy of 0.67. Figure 4b illustrates the most important features for predicting rupture are age, aspect ratio, and the presence of atherosclerosis, followed by irregular configuration of the aneurysm, familial predisposition, and localization at the basilar artery. Further characteristics were neck width and a history of nicotine consumption. The evaluation using SHAP correlated with the most important features, such as the presence of atherosclerosis, localization at the basilar artery, and familial predisposition. In comparison, the aspect ratio could not be confirmed with SHAP.

## 4. Discussion

This study investigated which risk factors contribute to the development of intracranial aneurysms, both in general and especially to their recurrence and rupture, with an ML model. The most significant predictors of aneurysm recurrence identified were age, aneurysm size, arterial hypertension, and a history of nicotine consumption. Independent risk factors for early recurrence within six months were multiple aneurysms and localization at the basilar artery. For aneurysm rupture, key predictive features included advanced age, basilar artery localization, atherosclerosis, irregular aneurysm morphology, and familial predisposition.

This is the first study, apart from comparable ML applications in anterior or mixed aneurysm cohorts, to use the statistical tool of machine learning to investigate posterior circulation aneurysms exclusively and confirms previous study results based on conventional statistical tools [4,5,6,7,8].

The size of our patient cohort is within the mid-range compared to recent studies utilizing machine learning [27,28]. Compared to studies on anterior circulation aneurysms, this cohort included a higher proportion of female patients, which is consistent with findings on posterior circulation aneurysms [16,29,30]. Female gender thus appears to be a relevant risk factor. Additionally, 71.6% of patients were over 50 years old, confirming the well-established association between increasing age and aneurysm prevalence [31].

Furthermore, this study included only endovascularly treated aneurysms, excluding conservatively managed patients, which limits the applicability of the results to the broader aneurysm population.

Our findings support arterial hypertension as a significant risk factor for aneurysm formation and rupture, which is consistent with prior research [32,33]. While only slightly more than half of the patients reported a history of nicotine use, this factor remains a well-documented risk [7,34]. Moreover, we observed an inverse relationship between BMI and aneurysm prevalence, which is in line with previous studies [35,36]. A high proportion (40%) of patients presented with multiple aneurysms, potentially due to the overrepresentation of female patients, as multiple aneurysms have been associated with female gender, nicotine use, and advanced age [37]. Using SHAP, no large differences were observed; however, aspect ratio was not significant for rupture and nicotine consumption was not an important factor for recurrence.

Basilar artery aneurysms accounted for 52% of cases, which corresponds to earlier reports [38]. Importantly, these aneurysms were associated with early recurrence (<6 months), contrasting with findings by Leng et al., who did not find a significant association between localization and recurrence in a study including both anterior and posterior circulation aneurysms [39].

Aneurysm size classification followed the Unruptured Cerebral Aneurysms Study (UCAS) criteria, categorizing aneurysms as small (<5 mm), medium (5–10 mm), large (10–25 mm), and giant (>25 mm) [19]. In our cohort, 83.4% of aneurysms were <10 mm, aligning with prior studies [40]. A total of 41.6% of aneurysms had an irregular configuration, with 55.2% of these being ruptured. This distribution is balanced compared to Beck et al., who found that multilobulated aneurysms were twice as common in ruptured cases.

Recurrence occurred in 33% of patients, a rate that varies widely in the literature [39,41]. Our ML models identified key predictors of recurrence at six and twelve months. A 2022 study demonstrated that machine learning could predict recurrence with a sensitivity of 81.2% and a specificity of 70.4%, which is roughly in line with our results [42]. The best ML model in that study showed an AUC of 0.84, which is better than our model (AUC 0.68), potentially due to the larger patient population. However, Lin et al. focused solely on recurrence prediction without considering individual risk factors, and there is currently no comparable ML-based study incorporating such factors.

For early recurrence (<6 months), the most influential predictors were age, presence of multiple aneurysms, dome-to-neck ratio, cardiovascular risk factors (nicotine use, hypertension), and basilar artery localization. For recurrences at follow-up (up to 12 months), aneurysm height, age, and nicotine use were the most significant features, while the overall recurrence rate was most associated with age, aneurysm width, and hypertension.

The primary predictors for aneurysm rupture were advanced age, aspect ratio, atherosclerosis, irregular aneurysm configuration, familial predisposition, neck width, and nicotine use. Basilar artery localization was also identified as a risk factor for rupture, though this may be influenced by the high proportion (52%) of basilar aneurysms in our cohort. Our findings on rupture are consistent with previous studies, which showed that irregular morphology and size ratio are significant factors [28,43].

This study represents a single-center cohort of posterior circulation aneurysms treated by an experienced interventional radiology team. Given the scalability of machine learning and the lack of comparable studies, our findings serve as a foundation for further research.

Nonetheless, several limitations apply to the present study. Being retrospective and exploratory in nature, the models, although consistent with previous findings, require validation in an external, prospective cohort to establish generalizability and clinical robustness. In addition, the heterogeneity of treatment techniques complicates generalizability. The clinical utility is limited due to the moderate AUCs and requires further research before clinical implementation. Although 10-fold cross-validation was rigorously applied to mitigate overfitting, the limited sample size and absence of an independent test set mean that overly optimistic and biased results cannot be excluded, potentially leading to diminished performance on unseen data. This concern is further underscored by the wide confidence intervals observed, revealing limited statistical power of the data set. Furthermore, model selection was based on achieving the highest AUC, which was chosen as a trade-off metric balancing sensitivity and specificity. While models with higher sensitivity or specificity could be identified, the use of AUC prioritized a balanced performance across both metrics. Moreover, as with many ML approaches, extreme caution is warranted in interpreting the results due to the lack of casual inferences. For clinical application, model results must be explainable. While both permutation importance and SHAP analysis were conducted and showed general agreement, their results cannot be fully trusted. Currently, no rigorous method exists to guarantee complete interpretability, and different methods might yield varying or even conflicting explanations, which remains a critical challenge for clinical adoption. In addition, although a range of feature selection methods was applied, the issue of correlated features such as dome height, dome width, and aneurysm size cannot be entirely resolved, highlighting the difficulty of disentangling multicollinearity in a causal sense.

Successful integration of ML algorithms into clinical routine requires demonstrable support, facilitation, and enhancement of physicians’ tasks. In the present model, ML algorithms were applied to identify risk factors. Such an approach could be interfaced with the hospital information system (HIS) to assist in the counseling and management of patients with neurovascular disease, thereby augmenting clinical decision-making. Clinical implementation appears feasible, as prediction software or ML-based models may be applied in genetically predisposed patients to guide screening strategies.

## 5. Conclusions

Aneurysms of the posterior cerebral circulation exhibit a poorer prognosis in the event of rupture compared to those in the anterior circulation. Additionally, the risk of recurrence and recurrent hemorrhage is significantly higher. This study utilizes machine learning techniques to analyze a cohort of patients and demonstrates that the majority were female and over 50 years of age, with a notable prevalence of arterial hypertension and a history of nicotine use. Among the identified risk factors for recurrence, age and aneurysm size, along with arterial hypertension and nicotine consumption, were the most significant. Moreover, the presence of multiple aneurysms and aneurysms localized to the basilar artery were found to be strong predictors of early recurrence within six months.

In terms of rupture risk, the best-performing model identified age, atherosclerosis, irregular aneurysm morphology, and familial predisposition as the most relevant factors. Basilar artery localization was also associated with an increased risk of rupture.

This study, to our knowledge, represents the first machine learning-based investigation into rupture risk and recurrence rates of both ruptured and unruptured intracranial aneurysms exclusively of the posterior cerebral circulation. The findings provide a valuable foundation for future research before clinical implementation in this area. In this case, the aim is to treat patients with aneurysms and associated risk factors before a fatal event occurs. Furthermore, there is a need for external validation in larger, prospective cohorts and the elimination of any selection bias that may exist.

## Figures and Tables

**Table 1:** Overview of all feature selection methods used. Filtering methods assign a score to each feature, while wrapper methods use a classifier to identify the most relevant features. Although all methods work without hyperparameters (except for the LASSO, where the regularization parameter C was left at the default value), a choice must be made on how many features to select. This number was treated as a hyperparameter and was tuned using a grid search.

Feature Selection	Type	Hyperparameters
Analysis of Variance (ANOVA)	Filtering	-
Bhattacharyya	Filtering	-
Fisher Score	Filtering	-
Least Absolute Shrinkage and Selection Operator (LASSO)	Wrapper	Regularization parameter C = 1
Mutual Information (MIM)	Filtering	-
Minimum Redundancy, Maximum Relevance Ensemble (MRMRe)	Filtering	-
None	Filtering	-
Pearson Correlation	Filtering	-
ReliefF	Filtering	-
t-Score	Filtering	-

**Table 2:** Overview of all classifiers used. Except for the Naïve Bayes method, all classifiers had hyperparameters, which were tuned using a grid search.

Classifier	Hyperparameters
Logistic Regression	Regularization parameter C in 2^(−6, −5, …, 5, 6)
Naïve Bayes	-
Neural Network (with three layers)	Number of neurons in Layers 1, 2, 3 in (2, 4, 8, 16, 32, 64)
Random Forest	Number of Trees in 50, 125, 250
Radial Basis Function-SVM (RBF-SVM)	Regularization parameter C and kernel parameter γ in 2^(−6, −5, …, 5, 6)

**Table 3:** Patient-related characteristics.

Characteristics	Total Patients (n = 229)
Gender [female]	164 (71.6%)
Age [years]	54.0 [18.0; 81.0]
Hypertension	166 (72.5%)
Nicotine consumption	126 (55.0%)
Atherosclerosis	57 (24.9%)
BMI [kg/m^2^]	
≤25	124 (54.1%)
25–30	69 (30.1%)
30–35	25 (10.9%)
35–40	6 (2.6%)
>40	5 (2.2%)

**Table 4:** Aneurysm-related characteristics.

Characteristics	Total Patients (n = 229)
Aneurysm localization [basilar artery]	119 (52.0%)
Aneurysm size—dome width [mm]	5.70 [1.50; 44.0]
Aneurysm size—dome height [mm]	6.40 [1.50; 24.6]
Aneurysm size—neck width [mm]	3.90 [0.90; 44.4]
Dome-to-neck ratio	1.50 [0.20; 4.60]
Aspect ratio	1.60 [0.20; 5.50]
Multiple aneurysms	88 (38.4%)
Irregular dome configuration—lobulation	96 (41.9%)

## Data Availability

The data is not available due to privacy and further research usage.

## Supplementary Materials

The following supporting information can be downloaded at https://www.mdpi.com/article/10.3390/diagnostics15182365/s1, Figure S1: AUC for predicting recurrence and rupture; Figure S2: SHAP values for predicting recurrence and rupture.

## Abbreviations

The following abbreviations are used in this manuscript:

ML	Machine Learning
SAH	Subarachnoid Hemorrhage
g	gram
RIS	Radiology Information System
BMI	Body Mass Index
PACS	Picture Archiving and Communication System
H&H	Hunt and Hess
GCS	Glasgow Coma Scale
DSA	Digital Subtraction Angiography
LASSO	Least Absolute Shrinkage and Selection Operator
ANOVA	Analysis of Variance
MIM	Mutual Information
MRMRe	Minimum Redundancy, Maximum Relevance Ensemble
SVM	Support Vector Machine
RBF	Radial Basis Function
ROC	Receiver Operating Characteristics
AUC	Area Under the Curve
SD	Standard Deviation
CI	Confidence Interval
kg	kilogram
m	meter
cm	centimeter
UCAS	Unruptured Cerebral Aneurysm Study
mm	millimeter
