# Correction: Növer et al. Prediction of Recurrence and Rupture Risk of Ruptured and Unruptured Intracranial Aneurysms of the Posterior Circulation: A Machine Learning-Based Analysis. *Diagnostics* 2025, *15*, 2365

**DOI:** 10.3390/diagnostics15232940

**Published:** 2025-11-21

**Authors:** Martin Növer, Hanna Styczen, Ramazan Jabbarli, Philipp Dammann, Martin Köhrmann, Tim Hagenacker, Christoph Moenninghoff, Michael Forsting, Yan Li, Isabel Wanke, Aydin Demircioğlu, Cornelius Deuschl

**Affiliations:** 1 Department of Anaesthesiology and Intensive Care Medicine, University Hospital Essen, Hufelandstrasse 55, 45147 Essen, Germany; 2 Institute of Diagnostic and Interventional Radiology and Neuroradiology, University Hospital Essen, Hufelandstrasse 55, 45147 Essen, Germany; 3 Department of Neurosurgery, University Hospital Essen, Hufelandstrasse 55, 45147 Essen, Germany; 4 Department of Neurology, University Hospital Essen, Hufelandstrasse 55, 45147 Essen, Germany; 5 Department of Radiology, Neuroradiology and Nuclear Medicine, Johannes Wesling University Hospital, Hans-Nolte-Strasse 1, 32429 Minden, Germany; 6 Swiss Neuroradiology Institute, Bürglistrasse 29, 8002 Zürich, Switzerland

In the original publication [1], Table 4 (Aneurysm-related characteristics) contains incorrect data, as values from Table 3 (Patient-related characteristics) were inadvertently transferred into it. Since these values do not correspond to the parameters intended for Table 4, the table requires revision. The corrected version of Table 4 appears below. The authors state that the scientific conclusions are unaffected. This correction was approved by the Academic Editor. The original publication has also been updated.

## Figures and Tables

**Table 4:** Aneurysm-related characteristics.

Characteristics	Total Patients (n = 229)
Aneurysm localization [basilar artery]	119 (52.0%)
Aneurysm size—dome width [mm]	5.70 [1.50; 44.0]
Aneurysm size—dome height [mm]	6.40 [1.50; 24.6]
Aneurysm size—neck width [mm]	3.90 [0.90; 44.4]
Dome-to-neck ratio	1.50 [0.20; 4.60]
Aspect ratio	1.60 [0.20; 5.50]
Multiple aneurysms	88 (38.4%)
Irregular dome configuration—lobulation	96 (41.9%)
