# Supplementary figures and images for: Prediction of Recurrence and Rupture Risk of Ruptured and Unruptured Intracranial Aneurysms of the Posterior Circulation: A Machine Learning-Based Analysis
Source: Diagnostics (Basel). 2025 Sep 17;15(18):2365. doi: 10.3390/diagnostics15182365 (PMC12468337; doi:10.3390/diagnostics15182365)

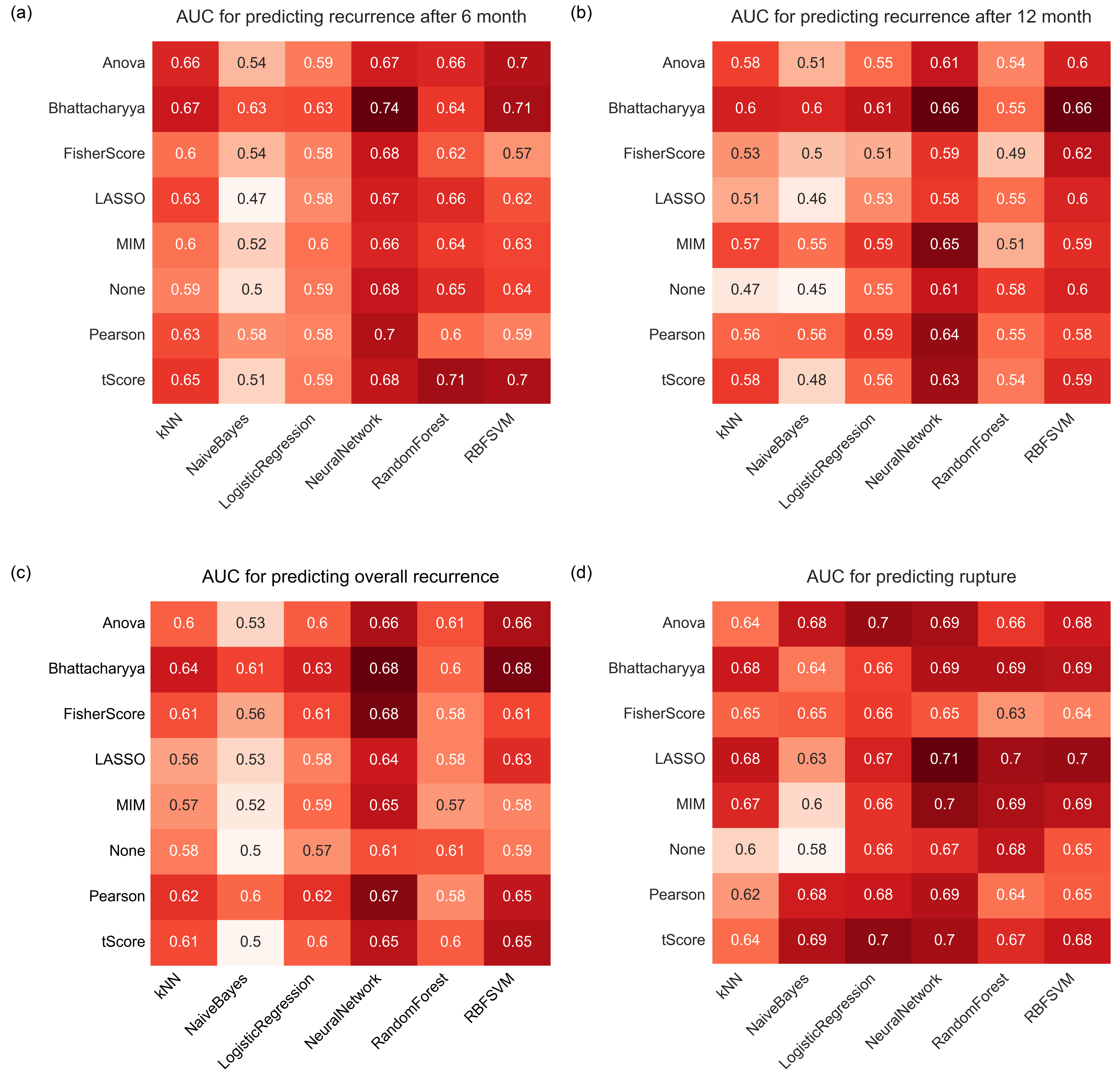

Supplement: Supplementary file 1 [file diagnostics-15-02365-s001.zip › paper/Figure_Results.png]

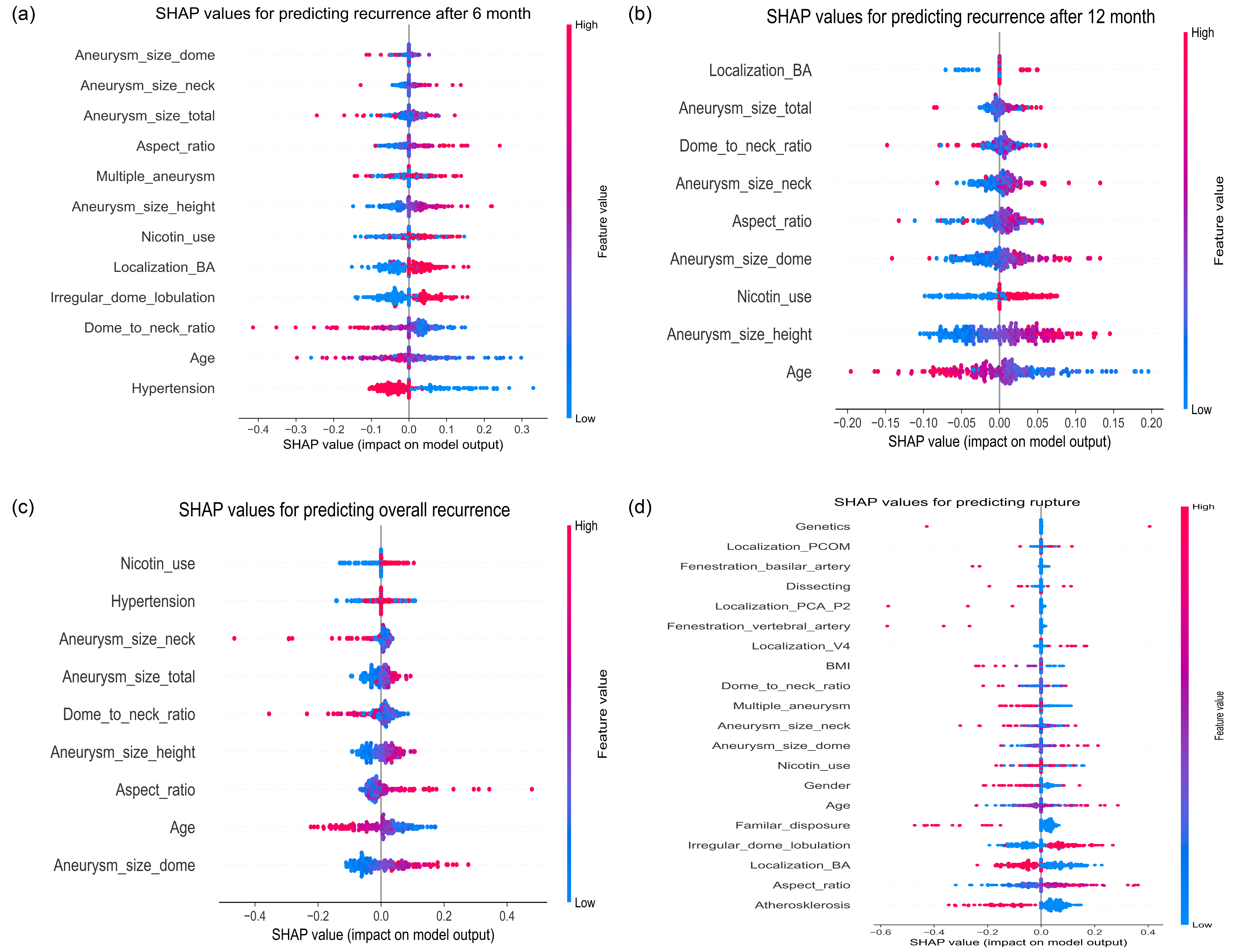

Supplement: Supplementary file 1 [file diagnostics-15-02365-s001.zip › paper/Figure_SHAP.png]
